# Supplementary material for: The Secretome of Parental and Bone Metastatic Breast Cancer Elicits Distinct Effects in Human Osteoclast Activity after Activation of β2 Adrenergic Signaling
Source: Biomolecules. 2023 Mar 30;13(4):622. doi: 10.3390/biom13040622 (PMC10135909; doi:10.3390/biom13040622)
Supplement: Supplementary file 1 [file biomolecules-13-00622-s001.zip › biomolecules-2219975-Supplementary Figures.pdf]

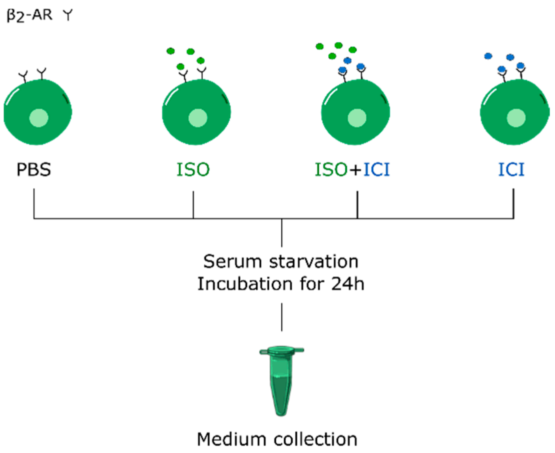

Figure S1. Conditioned medium collection from BC cell lines. β_2-AR expressing cell lines were incubated with either PBS, 1µM ISO (Green dots), 1µM ISO + 1µM ICI or 1µM ICI (Blue dots). Incubation was performed in reduced serum conditions for 24h, after which medium was collected and centrifuged.

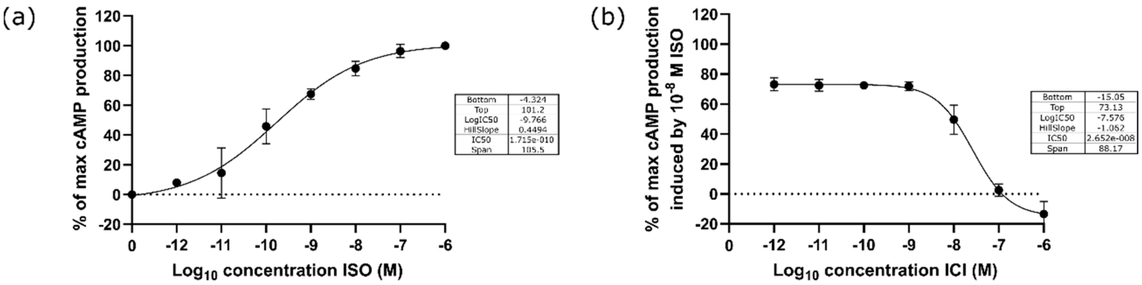

Figure S2. cAMP accumulation after ISO incubation. (a) ISO incubation leads to a dose-dependent accumulation of intracellular cAMP in β_2-AR expressing COS-7 cell lines, peaking at a concentration of 10^{-6}M. (b) The addition of ICI before and simultaneously to ISO inhibits the accumulation of cAMP, completely abrogating cAMP accumulation at a concentration of 10^{-6}M.

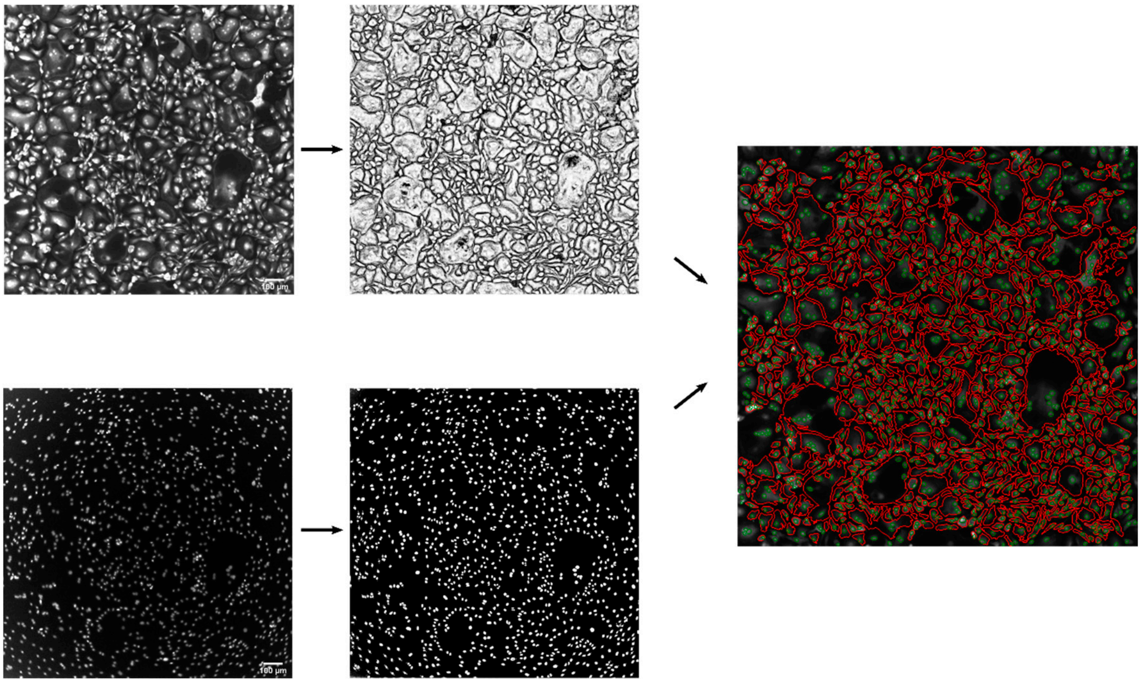

Figure S3. Automatic quantification of osteoclast differentiation. Raw images of HCS Cell Mask (top left) and DAPI (bottom left) are used to train the Ilastik toolkit. Automatic pixel classification is performed leading to the generation of probability masks either for the cell cytoplasm (top middle) or nuclei (bottom middle). Together with the raw images, the probability masks are loaded into a CellProfiler pipeline that automatically segments both the cytoplasm (right column, red) and the nuclei (right column, green) and generates excel files with the total number of osteoclasts and correspondent number of nuclei detected in the images.

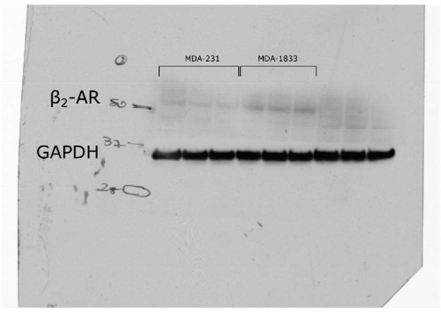

Figure S4. β2-AR protein expression in MDA-231 and MDA-1833 cells. Uncropped Western Blot of the expression of β2-AR on BC cell lines.

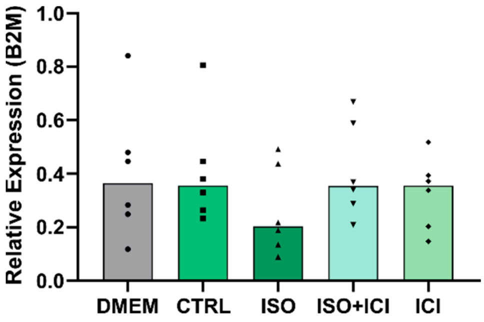

Figure S5. Osteoclast specific cathepsin K gene expression quantification. Pre-osteoclasts were treated with secretome of MDA-231 cells at day 5 of culture, and further cultured for another 4 days. The experiment was ended after fully differentiation of the osteoclasts at day 9, after which cells were lysed and mRNA was collected and purified. Data is expressed as median of individual data points from 6 independent experiments (Friedman's test).

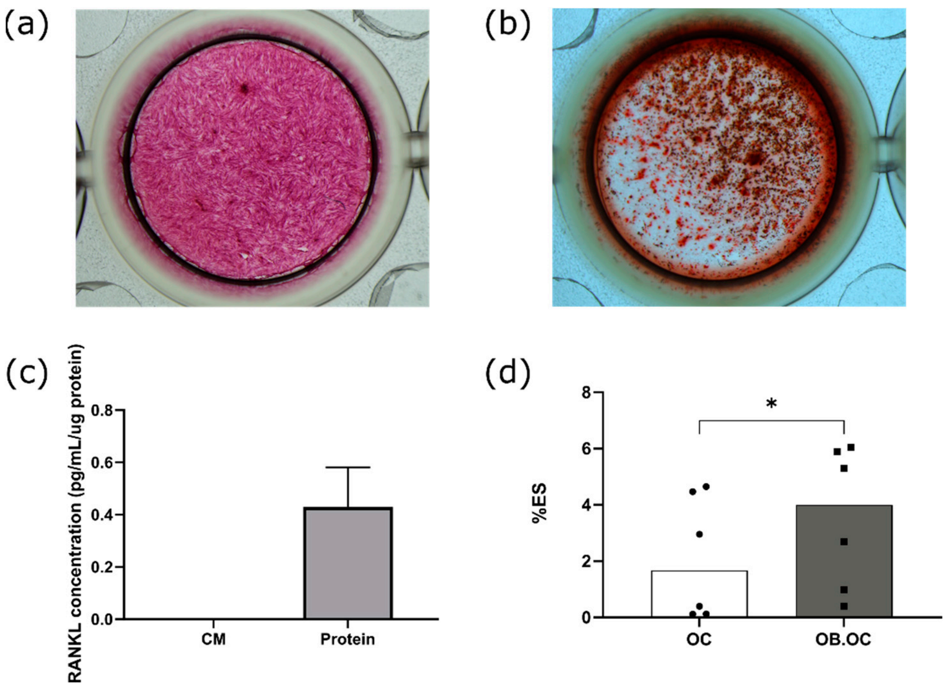

Figure S6. Cells obtained by trabecular bone outgrowth are from the osteoblast lineage. (a) After 7 days of culture, osteoblasts are positive for ALP. (b) After 14 days of culture, multiple calcium deposits are visible through alizarin red staining. Cells were cultured in 96-well plates, the whole well is shown. (c) RANKL quantification by ELISA. No RANKL was detected in the conditioned medium of osteoblasts, while most of it was detected in the cell lysates, suggesting that these cells express RANKL in the cell surface. Data from 2 independent experiments. (d) Comparison of osteoclast resorption activity between osteoclast monocultures and osteoblast/osteoclast co-cultures. Data is expressed as individual data points from 6 independent experiments (Friedman's test followed by Dunn's multiple comparisons test, *p≤0.05).

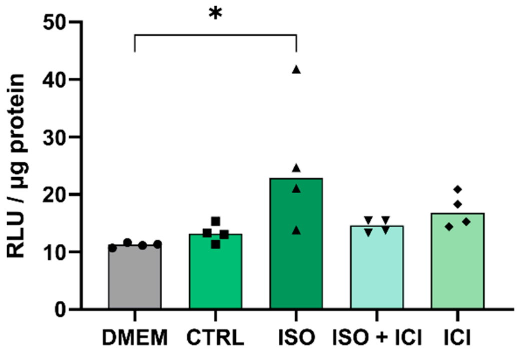

Figure S7. RANKL promotor activation in murine bone marrow stromal cells after incubation with MDA-231 conditioned medium. Bone marrow stromal cells express a luciferase reporter under RANKL promotor, and luminescence is quantified after incubation. Data is expressed as individual data points from 4 independent experiments (One Way ANOVA with Šidák's multiple comparison test, *p≤0.05).

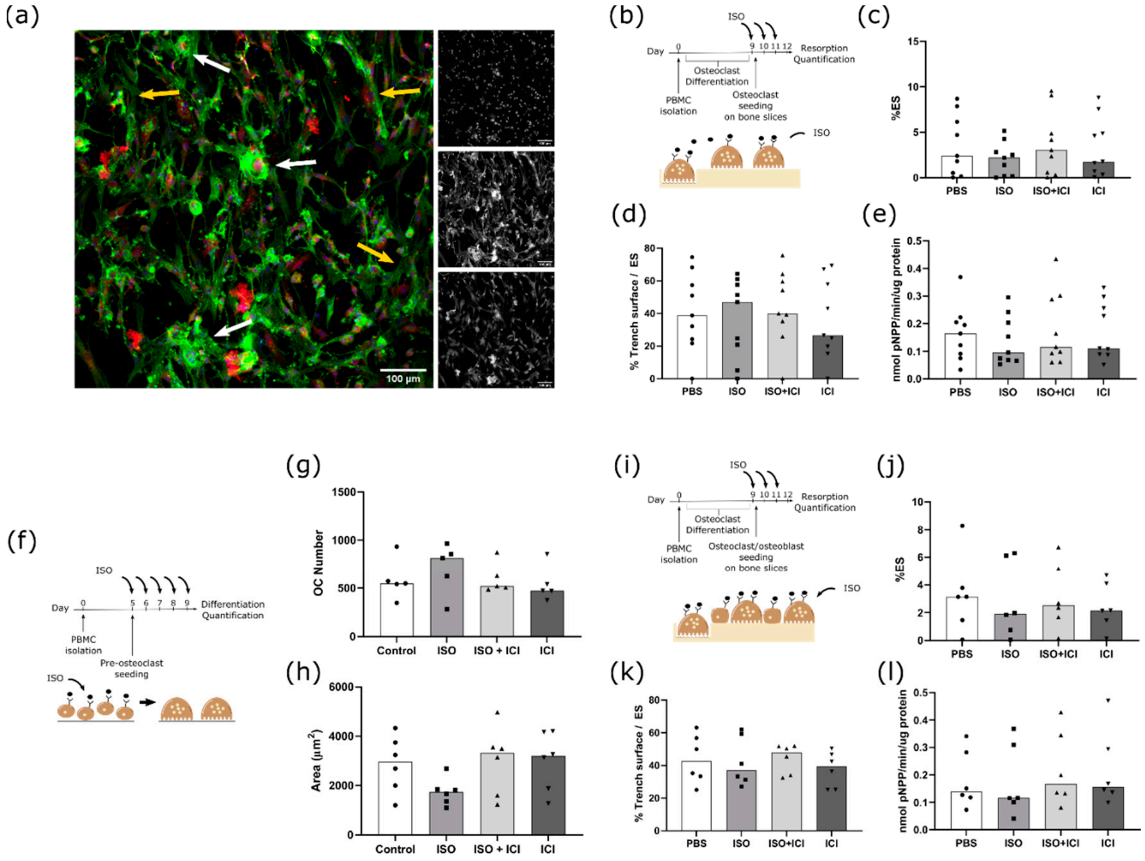

Figure S8. Direct effect of ISO on osteoclast differentiation and resorption activity. (a) Expression of β_2-AR on osteoclasts (white arrows) and osteoblasts (yellow arrows). DAPI (blue), F-Actin (green) and β_2-AR (red). Scale bar - 100µm. Inset single channel images are shown on the right: DAPI (top), F-Actin (mid) and β_2-AR (bottom). Inset scale bar – 100 µm. (b) Timeline of the resorption assay. Osteoclasts are differentiated for nine days and seeded on top of bone slices, followed by daily incubation with ISO for another three days. (c) Estimated percentage of eroded surface, (d) trench percentage per total eroded surface and (e) TRAcP activity quantified at the end of the experiment. Data is expressed as median of individual data points from 6 independent experiments (Friedman's test). (f) Timeline of the osteoclast differentiation assay. Pre-osteoclasts are seeded after five days of culture and stimulated daily with ISO for four more days, refreshing the media at day 7. (g) Osteoclast number and (h) area after four days of MDA-231 conditioned media stimulation. Osteoclasts with three or more nuclei were included in the analysis. Data is expressed as median of individual data points from 6 independent experiments (Friedman's test). (i) Timeline of the resorption assay in a co-culture setting. Mature osteoclasts were seeded with osteoblasts and stimulated daily with ISO for three days. (j) Estimated percentage of eroded surface, (k) trench percentage per total eroded surface and (l) TRAcP activity quantified at the end of the co-culture experiment. Data is expressed as median of individual data points from 6 independent experiments (Friedman's test).

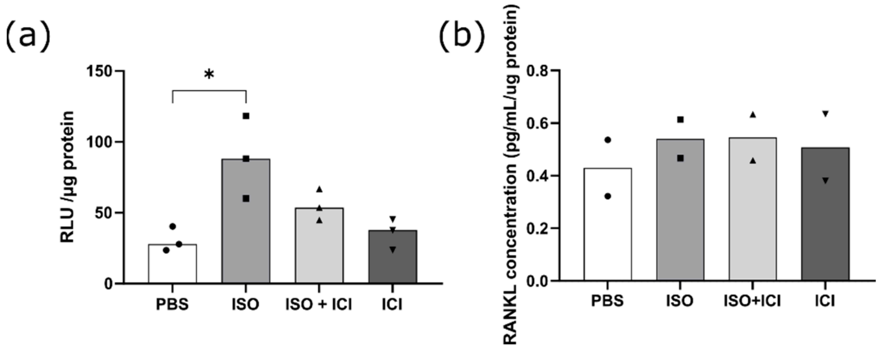

Figure S9. Direct effect of ISO on osteoblasts. (a) RANKL promotor activation in murine bone marrow stromal cells after incubation with MDA-231 conditioned medium. Bone marrow stromal cells express a luciferase reporter under RANKL promotor, and luminescence is quantified after incubation. Data is expressed as individual data points from 3 independent experiments (One Way ANOVA with Šidák's multiple comparison test, *$p \leq 0.05$). (b) Effect of ISO on the RANKL production in human osteoblasts, detected by ELISA. Data is expressed as individual data points from 2 independent experiments.

Supplementary Materials and Methods

cAMP accumulation assays

COS-7 (ATCC, Cat# CRL-1651, RRID:CVCL_0224) cells were cultured at 10% CO2 and 37 °C in DMEM 1885 supplemented with 10% FBS, 2 mM glutamine, 180 units/ml penicillin, and 45 g/ml streptomycin. The cells were transfected using the calcium phosphate precipitation method. To measure cAMP accumulation, the DiscoverX HitHunter cAMP assay was performed according to the manufacturer's protocol (DiscoverX, Fremont, CA, Cat#90-0075LM2). One day before the assay, the transiently transfected COS-7 cells expressing the human β2-AR were seeded into a 96 well white culture plate (35,000 cells/well, Perkin Elmer). On the assay day, the cells were washed with 1xHBSS and subsequently incubated in 1mM IBMX (Cat#410957) diluted in 2xHBSS for 30 minutes at 37 °C. Five µL of ligand (either agonist dose-response using increasing concentration of ISO or increasing concentration of the antagonist ICI together with a constant concentration of ISO (corresponding to ~80% activation)) were then added and the plate was incubated for 30 minutes at 37 °C. After the incubation, the assay medium was removed from the plate; the cells were washed with PBS and afterwards treated with ED/Lysis/CL and cAMP antibodies for 60 minutes, before EA solution was added. After a 3-hour incubation in the dark, the accumulation of cAMP was measured as luminescence using the Perkin Elmer EnVision 2104 Multilabel Microplate Reader. cAMP data was interpolated to the standard curve according to protocol. The non-linear regression curve fitting program Graphpad 8 was used to analyze the cAMP data and to obtain EC50 and Emax (Graphpad software, San Diego, CA).

ALP and Alizarin Red staining

For ALP staining, osteoblasts were cultured in 96 well plates at a density of 12 500 cells/well for 7 days in DMEM Low Glucose supplemented with 10% FBS, 50 µg/mL ascorbic acid, 10 mM β-glycerophosphate and 10 nM dexamethasone, changing medium at day 3. In the end of the experiment, culture medium was removed, cells were washed with PBS and were then fixed for 10 min in 4% PFA at RT. Osteoblasts were then washed in deionized water for 1 min, followed by another washing step for 15 min at RT. Cells were incubated with a staining solution (Fast Violet [Sigma-Aldrich, Cat#851-10CAP] and 0.25% Naphtol [Sigma-Aldrich, Cat#855] solution at a ratio of 25:1) for 45 min at RT in the dark. Osteoblasts were washed twice with deionized water and the full well was imaged with an SZX10 stereomicroscope (Olympus) coupled to an EP50 camera (Olympus).

For Alizarin Red staining, osteoblasts were cultured as described above for 14 days, changing medium twice a week. In the end of the experiment, culture medium was removed and cells were washed twice with cold PBS. Osteoblasts were then fixed in ice-cold 70% ethanol for 1 h at -20ºC. Cells were allowed to air-dry and were then washed twice with deionized water, followed by incubation in 2% Alizarin Red

(Sigma-Aldrich, Cat#A5533) solution in water (pH corrected to 4.2) for 15 min under gentle agitation at RT. Cells were washed twice with deionized water and the full well was imaged with an SZX10 stereomicroscope (Olympus) coupled to an EP50 camera (Olympus).

RANKL ELISA

RANKL protein levels in the lysate or conditioned media of osteoblasts was quantified by ELISA (Abcam, Cat#ABCAAB213841-96) according to the manufacturer's instructions.

Cathepsin K RT-PCR

Cell culture, RNA extraction, cDNA synthesis and RT-PCR were performed as described in the main Materials and Methods section. For the RT-PCR, specific primers were used for cathepsin K (FW 5'-TTCCCGCAGTAATGACACCC -3'; RV 5'- GGAACCACACTGACCCTGAT -3').

RANKL promotor activation quantification

UAMS-32-P (UAMS, RRID:CVCL_A8QR) murine bone marrow stromal cells stably transfected with a construct containing the entire murine RANKL gene coupled to luciferase as a reporter gene were kindly provided by Dr. Charles O'Brien (Center for Osteoporosis and Metabolic Bone Diseases, University of Arkansas for Medical Sciences, Little Rock, AR). These cells enable RANKL expression quantification through luciferase activity measurement in the conditioned medium of UAMS. UAMS were expanded in α-MEM (Gibco, Cat#12800-017) supplemented with 10% FBS and 1% pen/strep (α-MEM complete) and medium was changed every 2-3 days, until 80% confluency.

For luciferase activity quantification, UAMS were detached with 0.25% trypsin, centrifuged at 300g for 5 min and seeded in 24 well plates (Orange Scientific) at a density of 40 000 cells/well. Cells were left to adhere and proliferate for 24h, after which media was changed to α-MEM complete supplemented with BC conditioned media at a 1:1 ratio and cells were incubated for another 24 h. In the case of direct β_2-AR stimulation without BC secretome, 1µM ISO was added to the culture medium instead (as a control, β_2-AR activity was blocked with 1µM ICI prior and simultaneously to ISO to ensure complete blockage of the signaling). UAMS were then washed with PBS and incubated with 100µL of 1x reporter lysis buffer (Promega, Cat#PROME40300001) to promote cell lysis. Lysates were collected and frozen at -80°C for at least 24 h. Cell lysates were then centrifuged at 12 000 g to pellet cellular debris and the supernatant was stored at -80°C until further use. 20µL of cell lysate from each condition and 100µL of reconstituted luciferase assay reagent (Promega, Cat#PROME40300001) were added to a white/opaque 96 well plate. Luciferase activity was measured using a 2 s measurement delay followed by 10 s measurement read of absorbance at 655 nm in a Synergy Mx (BioTek) plate reader. The relative luminescence units (RLU) were normalized to the amount of protein in each sample (quantified using the DC Protein Assay (Bio-Rad) according to the manufacturer's instructions).

Immunocytochemistry

Osteoclasts and osteoblasts seeded on top of bone slices were washed twice with PBS and fixed with 4% PFA for 10 min. After another wash with PBS, cells were permeabilized with cold 1% (v/v) Triton X-100 in PBS for 5 min. Unspecific binding was then blocked with 0.1% BSA solution in PBS for 1 h at 37°C. Cells were incubated with a polyclonal rabbit anti-β_2-AR in a dilution of 1:100 in 0.1% BSA/PBS solution overnight at 4°C. Excess antibody was washed with PBS and cells were then incubated with donkey anti-rabbit AlexaFluor 568 (1:1000 dilution) together with FlashPhalloidin 488 (BioLegend) for 1 h at RT. After washing excess antibody with PBS, cells were counterstained with DAPI solution in PBS (1:1000 dilution) for 5 minutes. Samples were kept in PBS at 4°C in the dark until image acquisition. Images were acquired in a SP5 confocal microscope (Leica). Representative images were taken with a 10x objective at a resolution of 1024x1024 pixels. Brightness was adjusted with ImageJ software.

Direct β_2-AR activation in osteoclasts and osteoblasts

Osteoclast differentiation assays were performed as described in the main Materials and Methods section. Instead of BC secretome, pre-osteoclasts were stimulated at day 5 of culture with 1µM ISO or,

in the case of specific β_2-AR activation blockage, with 1µM ICI prior and simultaneously to the addition of 1µM ISO. Stimulation was performed daily until the end of the experiment.

Similarly, resorption assays in osteoclast monocultures or osteoclast/osteblast co-cultures were performed as described in the main Materials and Methods section. Instead of BC secretome, osteoclasts were stimulated at day 9 of culture with 1µM ISO or, in the case of specific β2-AR activation blockage, with 1µM ICI prior and simultaneously to the addition of 1µM ISO. Stimulation was performed daily until the end of the experiment.
