# Supplementary material for: The Secretome of Parental and Bone Metastatic Breast Cancer Elicits Distinct Effects in Human Osteoclast Activity after Activation of β2 Adrenergic Signaling
Source: Biomolecules. 2023 Mar 30;13(4):622. doi: 10.3390/biom13040622 (PMC10135909; doi:10.3390/biom13040622)
Supplement: Supplementary file 1 [file biomolecules-13-00622-s001.zip › biomolecules-2219975-Supplementary Tables.pdf]

Table S1. Description of the cohort of BC patients.

		Median age (min-max)	Primary Tumor	Molecular Subtype	Menopausal Status	Distant metastasis Site at Diagnosis
Healthy Donors		57 (46-65)	N/A	N/A	N/A	N/A
	Donor 1	62	N/A	N/A	N/A	N/A
	Donor 2	58	N/A	N/A	N/A	N/A
	Donor 3	57	N/A	N/A	N/A	N/A
	Donor 4	48	N/A	N/A	N/A	N/A
	Donor 5	62	N/A	N/A	N/A	N/A
	Donor 6	46	N/A	N/A	N/A	N/A
	Donor 7	56	N/A	N/A	N/A	N/A
	Donor 8	65	N/A	N/A	N/A	N/A
	Donor 9	56	N/A	N/A	N/A	N/A
	Donor 10	51	N/A	N/A	N/A	N/A
	Donor 11	64	N/A	N/A	N/A	N/A
	Donor 12	57	N/A	N/A	N/A	N/A
Stage I BC patients		64 (45-71)	T1	Luminal-like		N/A
	Patient 1	45	T1	Luminal B-like	Premenopausal	N/A
	Patient 2	61	T1	Luminal B-like	Postmenopausal	N/A
	Patient 3	66	T1	Luminal B-like	Postmenopausal	N/A
	Patient 4	68	T1	Luminal A-like	Postmenopausal	N/A
	Patient5	71	T1	Luminal A-like	Postmenopausal	N/A
	Patient 6	69	T1	Luminal B-like	Postmenopausal	N/A
	Patient 7	68	T1	Luminal B-like	Postmenopausal	N/A
	Patient 8	68	T1	Luminal B-like	Postmenopausal	N/A
	Patient 9	67	T1	Luminal B-like	Postmenopausal	N/A
	Patient 10	63	T1	Luminal B-like	Postmenopausal	N/A
	Patient 11	61	T1	Luminal A-like	Postmenopausal	N/A
	Patient 12	56	T1	Luminal A-like	Postmenopausal	N/A
Stage IV BC patients		55 (41-71)		Luminal-like		
	Patient 1	59	T3	Luminal B-like	Postmenopausal	Bone
	Patient 2	52	N/A	Luminal A-like	Postmenopausal	Bone
	Patient 3	64	N/A	Luminal B-like	Postmenopausal	Lung, Bone, Brain
	Patient 4	71	N/A	Luminal B-like	Postmenopausal	Bone, Liver, Lung
	Patient 5	51	T2	Luminal B-like	N/A	Skin, Lung, Bone
	Patient 6	57	T3	Luminal B-like	Premenopausal	Bone

Patient 7	53	N/A	Luminal B-like	Postmenopausal	Bone
Patient 8	51	N/A	Luminal B-like	Premenopausal	Bone
Patient 9	44	T3	Luminal A-like	Premenopausal	Bone, Lung
Patient 10	56	T4	Luminal B-like	Postmenopausal	Bone, Liver, Skin
Patient 11	58	T4	Luminal A-like	Postmenopausal	Bone
Patient 12	41	T3	Luminal A-like	Premenopausal	Bone

N/A – Not applicable/available

Table S2. Protein abundance ratio comparison between ISO treated MDA-231 and control MDA-231 samples

Gene Symbol	Abundance Ratio (ISOvsCTRL)	P value
CDH6	100	3,4E-16
MCFD2	100	3,4E-16
IGFBP1	2,976	3,4E-16
ALDOB	2,802	3,4E-16
MAN2B1	2,976	3,4E-16
STC1	2,652	3,4E-16
PLAU	2,568	3,4E-16
ABCA10	2,239	7,16E-12
VNN1	2,084	6,54E-10
FABP1	1,993	8,52E-09
TARS1	1,923	6,06E-08
BRD9	1,878	2,23E-07
IGFBP4	1,866	3E-07
PTK2B	1,754	4,9E-06
WASHC4	1,727	9,58E-06
PRC1	1,605	0,000169
CLU	1,596	0,000213
SERPINB4	1,566	0,000425
CTSL	1,508	0,001531
TPR	1,467	0,003897
HOOK3	1,466	0,00398
DDX39A	1,451	0,00539
CTSD	1,447	0,005945
C6	1,391	0,018738
PLAUR	1,382	0,02224
H2BC21	1,365	0,028902
SRGN	1,347	0,03991
IGFBP7	1,331	0,053154
GSTO1	0,01	3,4E-16
PBX4	0,01	3,4E-16
NCAN	0,035	3,4E-16
CPPED1	0,288	3,4E-16
SLC25A13	0,341	3,4E-16
TTN	0,386	3,4E-16
DSC1	0,404	6,42E-15
KRT10	0,52	5,62E-08
KRT9	0,534	2,23E-07
LMNA	0,56	1,95E-06
SERPINE1	0,588	1,76E-05
IGFN1	0,588	1,76E-05
KRT1	0,609	7,89E-05
MMP1	0,611	9,14E-05
NPM1	0,636	0,000337
CENPE	0,639	0,000404
MAN2A1	0,643	0,000495
KISS1	0,644	0,000527
CCN2	0,646	0,000604
ANXA2	0,654	0,000862
ARHGDIA	0,666	0,001608
COL6A2	0,677	0,002907
FBP1	0,679	0,003171
VIM	0,703	0,009364
EIF5AL1	0,708	0,011596
KRT14	0,718	0,016761
ADH5	0,723	0,020352
ATP5F1A	0,729	0,025211
TERT	0,731	0,02716

ANPEP	0,739	0,03368
GOT1	0,739	0,03429
AKAP13	0,741	0,035427
HADHB	0,741	0,03574
COL9A1	0,747	0,044673
PLXNB2	0,751	0,049436
VPS45	0,753	0,053683

Table S3. Protein abundance ratio comparison between ISO treated MDA-1833 and ISO treated MDA-231 samples.

Gene Symbol	Abundance Ratio (1833 ISO vs 231 ISO)	P value
VCP	100	2,91E-16
GSTO1	100	2,91E-16
SLC25A13	2,985	1,19E-11
DSC1	2,539	2,23E-08
PTX3	2,526	2,72E-08
PSAP	2,314	8,84E-07
KRT10	2,31	9,32E-07
VNN1	2,184	7,5E-06
MT1L	1,995	0,000141
KRT14	1,971	0,000142
PLVAP	1,956	0,000171
TPR	1,956	0,000173
RPL29	1,939	0,000221
ANPEP	1,921	0,000294
MCFD2	1,782	0,002194
PEPD	1,777	0,002374
DMBT1	1,753	0,003348
NACA	1,733	0,004404
KRT1	1,733	0,004408
LRRC8A	1,693	0,007781
PLG	1,671	0,01031
CCN2	1,642	0,014896
GRN	1,64	0,015265
IGF2	1,627	0,017886
OPA1	1,624	0,018592
NPM1	1,601	0,024787
FKBP1A	1,593	0,027353
TTR	1,589	0,028658
CPPED1	1,563	0,038659
KRT9	1,551	0,045396
TMEM132C	1,548	0,04683
RCN1	0,737	0,050725
MYOM2	0,727	0,038623
IGFBP4	0,724	0,036342
IGFBP3	0,723	0,035024
HGD	0,72	0,032832
TIMP2	0,719	0,031821
C4BPA	0,719	0,03205
ITIH2	0,714	0,027865
HBB	0,714	0,028367
CD44	0,708	0,023916
HLA-A	0,701	0,019793
OAF	0,679	0,01009
TPM1	0,676	0,009081
CTSZ	0,665	0,006297
APP	0,659	0,005054
DDX39A	0,657	0,004593
TNC	0,637	0,002221
FBP1	0,627	0,00146
IGFBP7	0,585	0,000214
CST3	0,581	0,000174
PTK2B	0,58	0,00017
MMP1	0,568	0,000134
PPIB	0,546	3,84E-05
THBS2	0,545	3,74E-05
SPARC	0,496	1,29E-06
SERPINE1	0,493	1,06E-06

STC1	0,491	8,84E-07
RNASET2	0,457	5,2E-08
VN1R5	0,45	2,72E-08
RPLP2	0,442	1,37E-08
IGFN1	0,438	8,98E-09
CTSL	0,418	1,09E-09
PLAUR	0,332	6,37E-15
IGFBP1	0,267	2,91E-16
LOX	0,237	2,91E-16
WASHC4	0,206	2,91E-16
SERPINB2	0,01	2,91E-16
SET	0,01	2,91E-16
